# 3D Clinical Overjet: Description of a Surface-Based Three-Dimensional Method for Measuring the Sagittal Relationship Between Dental Arches in Class II Malocclusion

**DOI:** 10.3390/jcm15176678

**Published:** 2026-08-28

**Authors:** Antonio Manni, Mauro Cozzani, Lorenzo De Marco, Giorgio Gastaldi, Fabio Castellana, Stefano Pera, Andrea Boggio

**Affiliations:** 1 Department of Dentistry, Vita-Salute San Raffaele University, Via Olgettina 58, 20132 Milano, Italy; dottantoniomanni@gmail.com (A.M.); maurocozzani@gmail.com (M.C.); lorenzodemarco93@gmail.com (L.D.M.); gastaldi.giorgio@hsr.it (G.G.); pe.ste.97@live.it (S.P.); 2 Istituto Giuseppe Cozzani, 19125 La Spezia, Italy; 3 Department of Interdisciplinary Medicine, University of Bari Aldo Moro, Piazza Giulio Cesare 11, 70100 Bari, Italy; castellanafabio@gmail.com

**Keywords:** clinical overjet, class II malocclusion, digital dental models, orthodontic diagnosis

## Abstract

**Objectives:** Conventional overjet is widely used to describe sagittal interarch relationships, but its measurement depends on predefined landmarks and acquisition methods. This study aimed to describe a standardized three-dimensional method, termed 3D Clinical Overjet, for measuring the minimum sagittal distance between anterior dental surfaces in Class II malocclusion. **Methods:** This retrospective methodological study applied the proposed protocol to 42 consecutive growing patients with skeletal and dental Class II malocclusion. Digital intraoral scans obtained at baseline (T0) and immediately before mandibular advancement (T1) were analyzed by a single examiner. 3D Clinical Overjet was defined as the minimum linear distance, measured parallel to the occlusal plane, between the labial surface of any mandibular incisor and the palatal surface of the nearest maxillary incisor. No intra- or inter-examiner reliability assessment was performed because the study was designed to demonstrate feasibility rather than validate measurement properties. Associations with radiographic overjet were assessed using Spearman’s rank correlation; T0–T1 changes were analyzed using the Wilcoxon signed-rank test, and tooth-distribution changes were analyzed using a marginal homogeneity test. **Results:** 3D Clinical Overjet was obtained in all 42 subjects at T0 and in 41 at T1. Among the 41 subjects with paired observations, median 3D Clinical Overjet increased from 0.60 [0.10–2.00] mm at T0 to 1.70 [0.60–4.30] mm at T1 (W = 224, *p* = 0.054; rank-biserial correlation = 0.36). The maxillary tooth determining the minimum distance varied among patients and across time points. 3D Clinical Overjet was not significantly associated with radiographic overjet at T0 (Spearman ρ = 0.095, *p* = 0.558; *n* = 40). **Conclusions:** 3D Clinical Overjet is a feasible surface-based three-dimensional method for identifying the minimum sagittal distance across the anterior dentition without restricting measurement to a predefined incisor pair. In this sample, the tooth determining the minimum distance varied among patients and across time points, and 3D Clinical Overjet showed no significant association with radiographic overjet. These findings support the feasibility of the proposed geometric approach; its reproducibility, validity, and clinical relevance remain to be established.

## 1. Introduction

The assessment of the sagittal relationship between the dental arches represents one of the fundamental aspects of orthodontic diagnosis. Over the years, numerous parameters have been proposed to describe this relationship, each characterized by specific anatomical, geometric, and methodological assumptions. The choice of which parameter to use depends on the diagnostic objectives, the acquisition method, and the clinical context in which the measurement is performed [1,2].

Among the most widely used parameters, overjet has represented one of the fundamental references for describing the anterior sagittal relationship for decades. In addition to being a routine measurement in clinical practice, it is frequently used to assess the severity of malocclusions, monitor the effects of orthodontic treatment, and support treatment planning [1,2,3]. In particular, in Class II malocclusions, the magnitude of the overjet has often been considered an indicator of the severity of the sagittal discrepancy and has been used as a criterion for guiding treatment selection [3,4,5]. Specifically, Proffit and colleagues proposed including the magnitude of the overjet among the criteria for distinguishing cases suitable for orthodontic treatment from those requiring combined orthodontic-surgical treatment, and can contribute to the definition of so-called borderline cases [4,5]. These observations highlight the clinical relevance traditionally attributed to this parameter in orthodontic decision-making.

Despite its widespread use, the term overjet does not identify a universally defined measurement. The methods reported in the literature differ according to the clinical setting, the anatomical landmarks selected, and the acquisition technique employed. The measurement can be performed directly in the oral cavity, on dental casts, or on lateral cephalometric radiographs, using reference points that do not necessarily describe the same anatomical relationship. Consequently, measurements referred to by the same term may not be entirely comparable, sometimes making the interpretation and comparison of findings reported in the literature more difficult [1,2,3].

The introduction of intraoral scanners and three-dimensional digital dental models has profoundly expanded the possibilities for analyzing dental relationships. Unlike traditional methods, digital models allow for the simultaneous visualization of the entire morphology of the dental surfaces and enable measurements that are not necessarily constrained to predefined anatomical landmarks. Digital technologies therefore make it possible not only to measure established parameters with greater precision but also to explore new approaches for describing the anatomical relationships between the dental arches [6,7,8,9].

Although three-dimensional digital models have substantially expanded the possibilities for orthodontic measurements, previously described digital approaches to overjet have generally reproduced the conventional measurement using predefined dental landmarks or selected incisors. For example, digital overjet has been assessed between predetermined central incisors or by transferring conventional cast-based measurements to three-dimensional software environments [10,11,12]. These approaches have demonstrated that conventional overjet can be reliably measured on digital models, but their primary objective has remained the digital reproduction of an established parameter. In contrast, the method proposed in the present study changes the geometric target of the measurement: rather than measuring the distance between predefined incisors or landmarks, it searches across the anterior dental region for the minimum sagittal distance between opposing dental surfaces, independently of the particular teeth involved. To our knowledge, this specific surface-based approach has not previously been standardized for the assessment of anterior sagittal interarch relationships. 3D Clinical Overjet was developed to address this methodological gap. Thus, the methodological need addressed by 3D Clinical Overjet is not the ability to measure conventional overjet digitally, but the absence of a standardized surface-based approach for identifying the minimum sagittal distance across the entire anterior dentition. Despite the considerable advances achieved in the treatment of Class II malocclusions, the availability of parameters truly capable of predicting treatment outcomes remains limited. At the same time, modern orthodontics is increasingly oriented toward personalized, predictable treatments supported by objective measurements [1,2,13]. Moreover, orthodontic biomechanics may modify tooth inclination and consequently alter the anterior sagittal dental relationship, further supporting the potential value of a surface-based three-dimensional assessment [14]. In this context, the possibility of characterizing the sagittal relationship between the dental arches by means of novel three-dimensional parameters represents an area of potential interest.

The method proposed in the present study arises from these considerations and from the technological evolution. The underlying concept of the protocol is to identify the minimum sagittal distance between the anterior dental surfaces, regardless of which pair of incisors is involved. This measurement, referred to in the present study as 3D Clinical Overjet, is not intended to redefine the traditional concept of overjet but rather to propose a different way of describing the anterior sagittal relationship by taking advantage of the opportunities offered by three-dimensional digital dental models.

The biological rationale underlying the development of the proposed method derives from the biomechanics of functional mandibular advancement. Functional appliances correct Class II relationships by maintaining the mandible in an advanced position, with treatment effects resulting from variable combinations of skeletal and dentoalveolar adaptations. Previous studies have also shown that the amount of mandibular advancement, or bite jumping, may influence the magnitude of dentoalveolar changes, particularly at the mandibular incisors [13,14,15,16]. These observations suggest that the anterior dental relationship and the amount of sagittal clearance available during mandibular advancement may represent relevant biomechanical features of functional treatment. On this basis, we hypothesized that identifying the minimum sagittal distance between opposing anterior dental surfaces could provide a more anatomically specific description of this relationship than a measurement restricted to predefined incisors. However, no direct evidence currently demonstrates that this minimum distance represents the effective amount of mandibular advancement achievable in an individual patient. This assumption therefore constitutes the biological rationale for developing the method rather than a hypothesis tested in the present study. Therefore, the primary aim of the present study was to describe a standardized protocol for measuring 3D Clinical Overjet using three-dimensional digital dental models and to assess its feasibility when applied to a clinical sample of growing patients with Class II malocclusion. As secondary exploratory objectives, we described the distribution of the maxillary tooth determining the minimum sagittal distance, evaluated changes in 3D Clinical Overjet following orthodontic preparation, and explored its association with conventional radiographic overjet. The study was conceived as a methodological proposal and was not designed to establish the reliability, validity, diagnostic accuracy, or clinical usefulness of the proposed measurement.

## 2. Materials and Methods

### 2.1. Study Design

The present study describes a surface-based three-dimensional method for measuring the anterior sagittal relationship, referred to as 3D Clinical Overjet, and illustrates its application in a retrospective sample of patients with Class II malocclusion. The study was designed as a methodological feasibility study and not as a validation study; therefore, no reference standard was used to assess criterion validity or diagnostic accuracy. All measurements were performed by a single examiner. Intra-examiner repeatability, inter-examiner reliability, and measurement error were not assessed in the present study.

### 2.2. Sample Characteristics and Eligibility Criteria

The method was applied to 42 consecutive growing patients (22 females and 20 males) with skeletal and dental Class II malocclusion. At T0, the median age was 9.11 years [IQR: 8.36–10.84; range: 6.68–16.16 years]. Patients were evaluated at two time points: at the beginning of treatment (T0) and immediately before mandibular advancement (T1), after completion of the preparatory orthodontic phase. Baseline sagittal malocclusion severity was characterized using the ANB angle, Wits appraisal, and conventional radiographic overjet, as reported in Table 1. The inclusion criteria were: (1) skeletal Class II malocclusion ANB > 4°; (2) bilateral Class II canine relationship of at least one-half cusp; and (3) indication for orthopedic treatment by means of mandibular advancement. Patients with craniofacial syndromes, cleft lip and palate, previous orthodontic treatment, agenesis or loss of permanent anterior teeth, restorations capable of modifying incisal morphology, or incomplete diagnostic records were excluded.

To minimize selection bias, patients were screened consecutively according to the chronological order of the available clinical records during the study period, and the same predefined eligibility criteria were applied to all potentially eligible subjects. All patients meeting the eligibility criteria and having the required diagnostic records were included, without discretionary case selection based on 3D Clinical Overjet values or subsequent treatment response. No case matching or outcome-based selection was performed. Nevertheless, because availability of complete records was required in this retrospective study, residual selection bias cannot be excluded and is acknowledged as a study limitation.

Before functional therapy, all patients underwent maxillary expansion. Additional alignment and/or decompensation of the maxillary incisors was performed in 17 of 42 patients when clinically indicated. T1 was defined immediately after completion of the preparatory phase and before mandibular advancement. Because the preparatory procedures were not identical across subjects, T0–T1 changes were analyzed only as an overall exploratory comparison and were not intended to assess the effect of individual orthodontic procedures. No formal a priori sample size calculation was performed because the study was conceived as an exploratory methodological feasibility study rather than as a hypothesis-testing validation study. The sample consisted of all consecutive eligible patients with complete records available during the study period. Accordingly, the sample size should be considered pragmatic, and its limited size is acknowledged as a study limitation.

### 2.3. Ethics Approval

The study was approved by Comitato Etico Territoriale Lombardia 1 (Approval number STM24, June 2025), and all patients, or their legal guardians, had provided informed consent for the use of their clinical records for research purposes.

### 2.4. Measurement Protocol

Intraoral scans were acquired using a TRIOS 5 intraoral scanner (3Shape, Copenhagen, Denmark) according to the routine clinical acquisition protocol used for full-arch intraoral scanning. The scans were exported from the TRIOS system and imported directly into the SureSmile Advanced platform (Dentsply Sirona, Charlotte, NC, USA), which was used for all subsequent three-dimensional processing and measurements [6,7,8,9]. 3D Clinical Overjet was defined as the minimum linear distance, measured parallel to the occlusal plane, between the labial surface of any mandibular incisor and the palatal surface of the nearest maxillary incisor. Unlike conventional overjet, the measurement was not restricted to a predefined pair of incisors or anatomical landmarks, but was obtained by searching for the minimum sagittal distance across the entire anterior incisor region.

**Figure 1:**
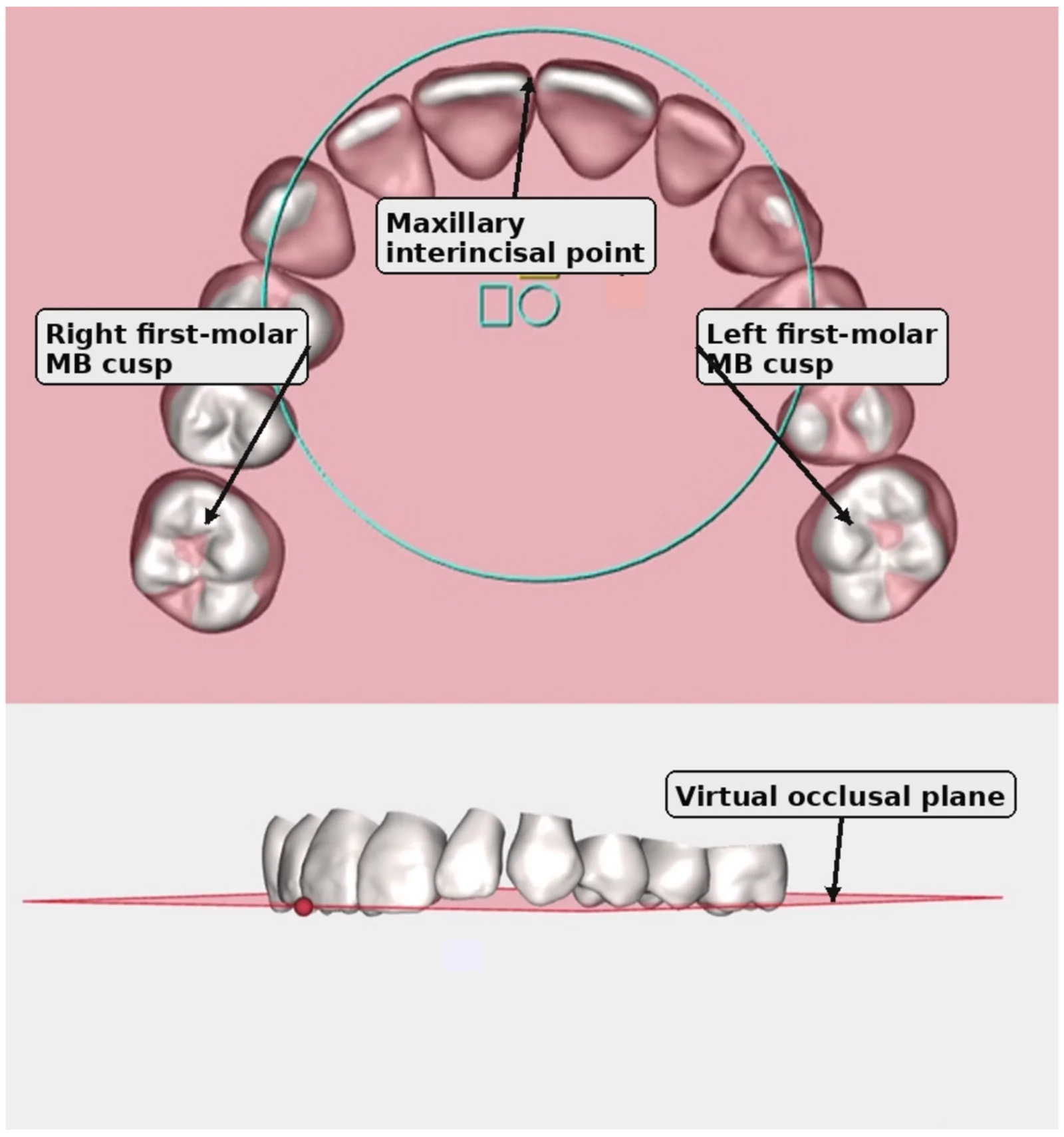
Construction of the virtual maxillary occlusal plane. The maxillary interincisal point and the mesiobuccal cusps of the right and left maxillary first molars are used as reference landmarks. The plane defined by these three landmarks serves as the reference for 3D Clinical Overjet measurement.

**Figure 2:**
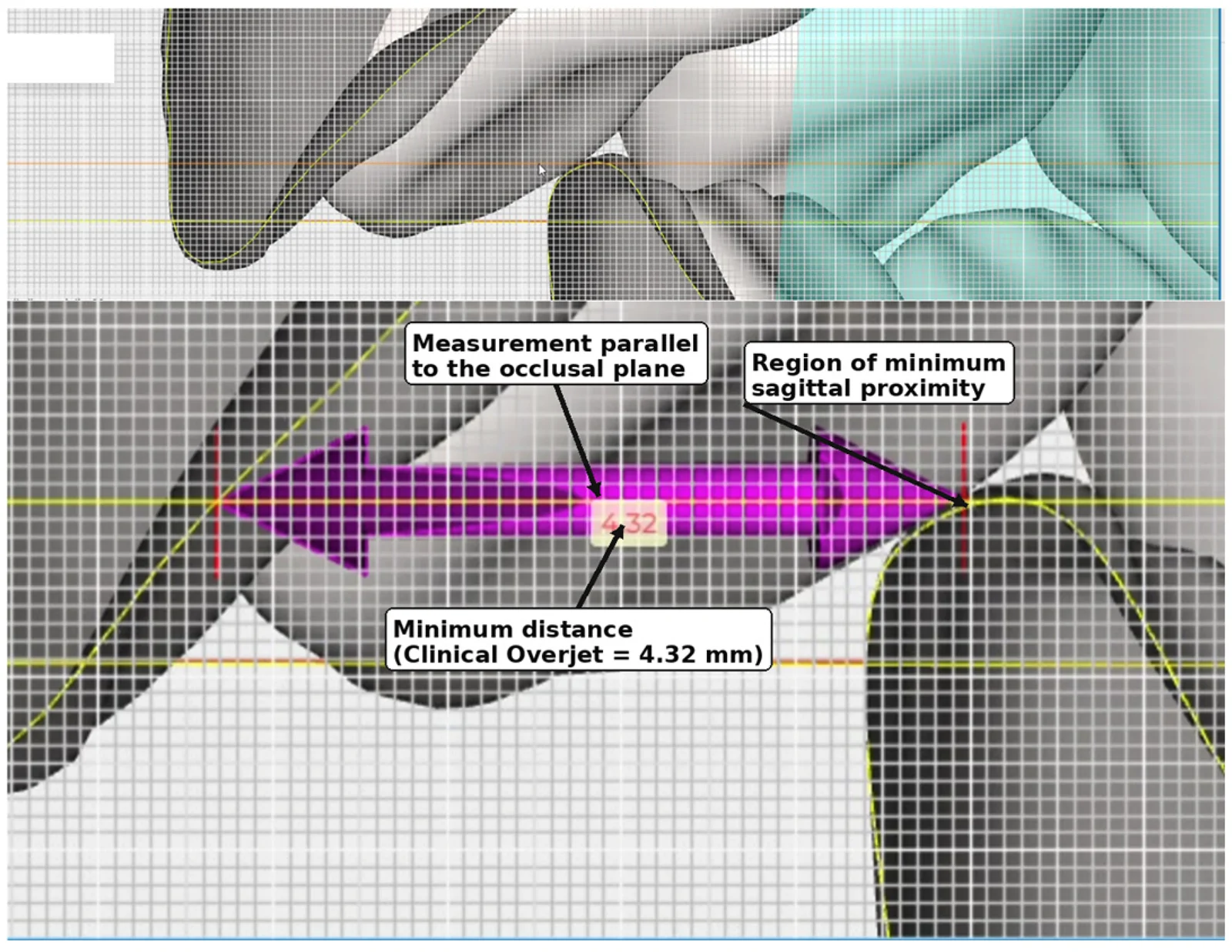
Measurement of 3D Clinical Overjet. After identification of the region of closest sagittal proximity between the opposing anterior dental surfaces, the linear measurement is performed parallel to the virtual occlusal plane. Measurement points are progressively repositioned across the opposing surfaces until the minimum sagittal distance is identified. In the example shown, the minimum distance, recorded as 3D Clinical Overjet, is 4.32 mm.

The measurement was performed according to the following standardized sequence:

(1) A virtual maxillary occlusal plane was constructed using the maxillary interincisal point and the mesiobuccal cusps of the right and left maxillary first molars (Figure 1);
(2) The anterior dentition was inspected to identify the region of closest sagittal proximity between opposing incisor surfaces;
(3) Within this region, linear measurements were performed parallel to the occlusal plane between the labial surface of the mandibular incisors and the palatal surface of the maxillary incisors;
(4) The measurement points were progressively repositioned across the opposing dental surfaces, and additional sections were examined whenever necessary to determine whether a smaller sagittal distance was present;
(5) The lowest value identified during this procedure was recorded as the 3D Clinical Overjet, together with the maxillary tooth involved in the minimum-distance measurement (11, 12, 21, or 22) (Figure 2).

For each patient, the conventional overjet was also measured on the lateral cephalometric radiograph and was defined as the horizontal distance between the incisal edge of the maxillary central incisor and the labial surface of the corresponding mandibular central incisor. Radiographic overjet was selected as the conventional comparator because it represents a routinely reported measurement in cephalometric assessment and in the orthodontic literature on sagittal relationships. It was used solely for exploratory comparison and was not considered a reference standard for validation of 3D Clinical Overjet. No separate chairside 3D Clinical Overjet measurement was used as a reference standard because the present study was not designed as a criterion-validation study. All measurements were performed by the same experienced examiner. Before data collection, the examiner underwent preliminary training to standardize the procedure for identifying and verifying the minimum sagittal distance according to the protocol described above. The examiner was not blinded to the observation time point, and no formal procedure for observer blinding was implemented. No formal intra- or inter-examiner reliability assessment was performed.

### 2.5. Statistical Analysis

Statistical analyses were performed using R software (Version 4.5.3. Vienna, Austria). Data were reshaped from wide to long format, with each row representing a single tooth-level observation nested within subject and time point (T0, T1); observations with missing values were excluded from the relevant analyses. Paired T0–T1 analyses were restricted to subjects with 3D Clinical Overjet measurements available at both time points.

Descriptive statistics: For each time point (T0, T1), the absolute and relative frequency of each tooth identified as the first contact point were calculated, using the number of distinct subjects assessed at that time point as the denominator. Results are presented as bar charts stratified by time point. Baseline and follow-up sample characteristics were summarized in a descriptive table stratified by time point.

Correlation analysis: For exploratory purposes, the association between 3D Clinical Overjet at T0 and conventional radiographic overjet measured at the same time point was assessed to investigate the degree of correspondence between the two measurements. Because the two methods use different anatomical and geometric definitions, this analysis was not intended as a validity or agreement assessment. The association was evaluated using Spearman’s rank correlation coefficient (ρ) because of the non-normal distribution of the variables. The correlation coefficient, corresponding *p*-value, and sample size are reported, and the relationship is illustrated graphically using a scatterplot.

Comparison between time points: Normality of the paired differences in 3D Clinical Overjet between T0 and T1 was assessed using the Shapiro–Wilk test. Changes in 3D Clinical Overjet between T0 and T1 were evaluated using the Wilcoxon signed-rank test for paired samples, restricted to subjects with observations available at both time points, because the distribution of paired differences deviated from normality. Changes in the distribution of the maxillary tooth determining the minimum sagittal distance between T0 and T1 were assessed in the paired sample using a marginal homogeneity test for paired categorical data. Continuous variables are reported as median and interquartile range (IQR). For effect-size and correlation estimates, 95% confidence intervals were obtained using non-parametric bootstrap resampling. A two-sided *p*-value <0.05 was considered statistically significant.

## 3. Results

### 3.1. Distribution of 3D Clinical Overjet

**Figure 3:**
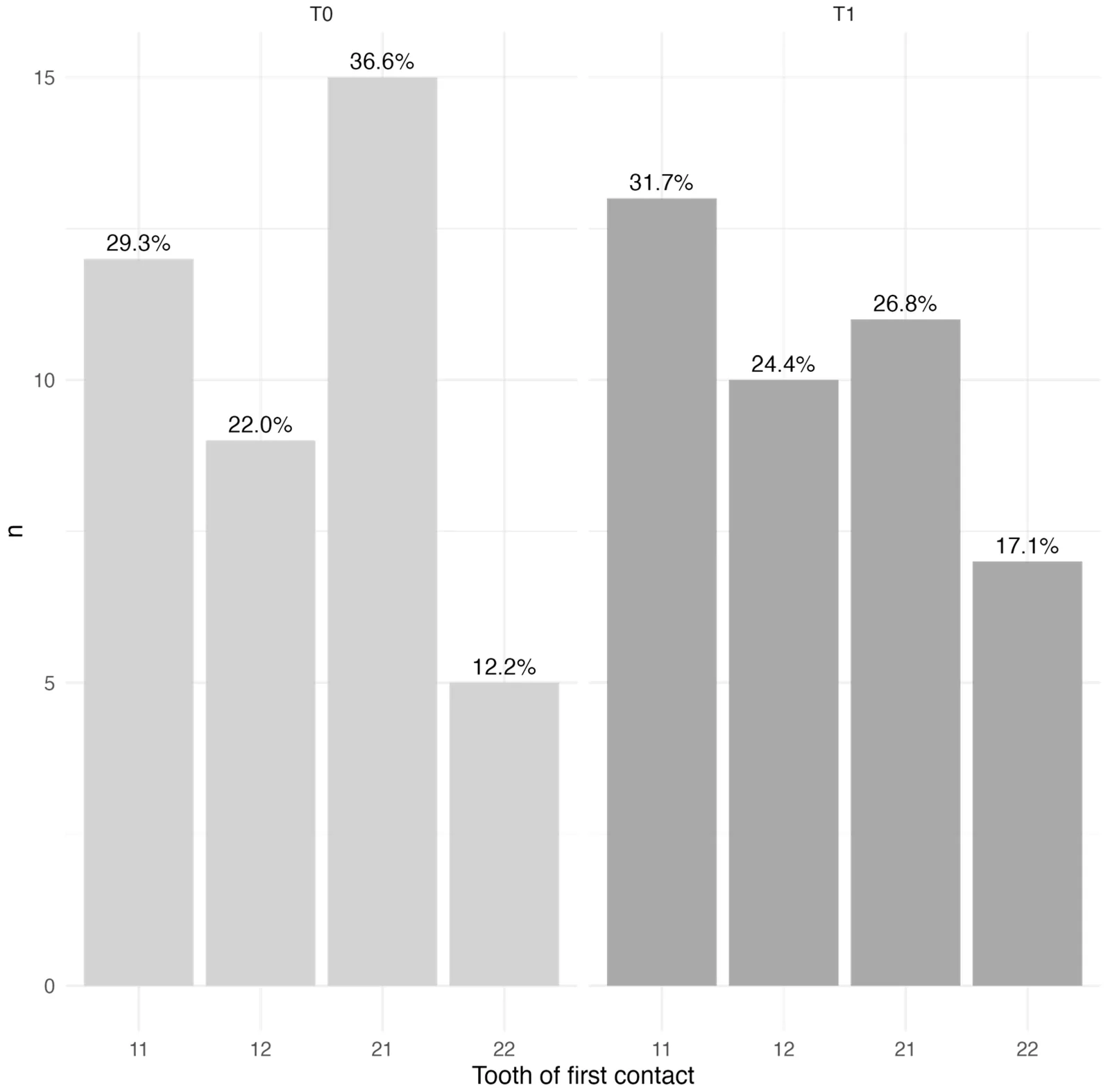
Distribution of the maxillary tooth determining the minimum sagittal distance at T0 and T1.

The study included 42 patients, comprising 22 females (52.4%) and 20 males (47.6%). At T0, the median age was 9.11 years [IQR: 8.36–10.84; range: 6.68–16.16], the median ANB angle was 5.85° [IQR: 5.23–7.00], and the median Wits appraisal was 3.00 mm [IQR: 2.00–4.00]. The median conventional radiographic overjet was 5.90 mm [IQR: 4.35–7.65]. Demographic and baseline sagittal characteristics are summarized in Table 1. 3D Clinical Overjet was successfully obtained for all 42 subjects at T0 and for 41 subjects at T1. Measurement time and technical difficulties were not systematically recorded because these aspects were not predefined outcomes of the retrospective study. The minimum sagittal distance between the anterior dental surfaces was therefore identified at baseline (T0) and immediately before mandibular advancement (T1), with paired T0–T1 analyses performed on 41 subjects. At T0, the minimum distance was most frequently located on the maxillary left central incisor (21; 36.6%), followed by teeth 11 (29.3%), 12 (22.0%), and 22 (12.2%). At T1, the distribution remained substantially stable, with a slight predominance of tooth 11 (31.7%), followed by teeth 21 (26.8%), 12 (24.4%), and 22 (17.1%). Among the 41 subjects with paired observations, the distribution of the maxillary tooth determining the minimum sagittal distance did not significantly change between T0 and T1 (marginal homogeneity test, *p* = 0.751) (Figure 3).

The median 3D Clinical Overjet was 0.50 [0.30, 1.97] mm at T0 and 1.70 [0.60, 4.30] mm at T1 (Table 1).

### 3.2. Exploratory Comparison with Conventional Overjet

**Figure 4:**
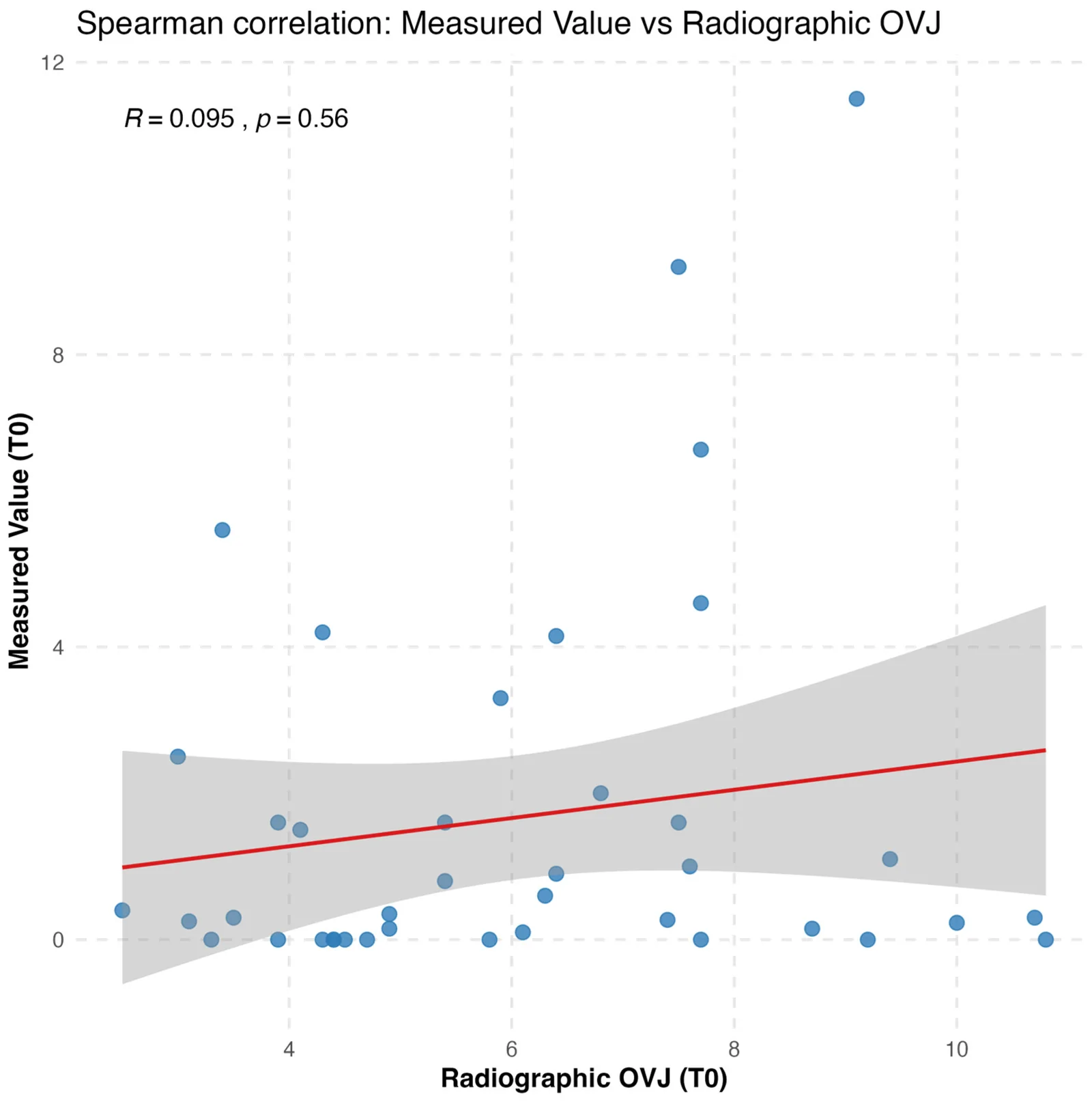
Spearman Correlation: Clinical (Measured) vs. Radiographic Overjet (Spearman ρ = 0.095, 95% CI −0.238 to 0.409, *p* = 0.558; *n* = 40).

The median conventional radiographic overjet at T0 was 5.90 mm [IQR: 4.35–7.65]. The exploratory analysis showed no significant association between 3D Clinical Overjet and conventional radiographic overjet at T0 (Spearman ρ = 0.095, 95% CI: −0.238 to 0.409, *p* = 0.558; *n* = 40). The scatterplot showed substantial variability between the two measurements (Figure 4).

### 3.3. Exploratory Comparison Between Time Points

Changes in 3D Clinical Overjet between T0 and T1 were evaluated in the 41 subjects with paired observations using the Wilcoxon signed-rank test. 3D Clinical Overjet increased from a median of 0.60 mm [IQR: 0.10–2.00] at T0 to 1.70 mm [IQR: 0.60–4.30] at T1. The median paired change was 0.25 mm [IQR: −0.12–1.85]; however, the difference did not reach statistical significance (W = 224, *p* = 0.054; rank-biserial correlation = 0.36, 95% CI: −0.03 to 0.67) (Table 2).

## 4. Discussion

The assessment of the sagittal relationship between the dental arches represents one of the fundamental aspects of orthodontic diagnosis. Although overjet is one of the most widely used parameters in clinical practice, its definition and measurement methods are not uniform and vary according to the diagnostic context, the anatomical landmarks adopted, and the acquisition technique employed [1,2,3]. The introduction of three-dimensional digital dental models now offers the opportunity to describe dental relationships using geometric approaches that cannot be achieved with conventional methods [6,7,8,9]. It is within this context that the method proposed in the present study was developed, with the aim of identifying and measuring, in a standardized manner, the minimum sagittal distance between the anterior dental surfaces.

Conventional clinical and cast-based overjet measurements are simple, widely established, and directly applicable in routine orthodontic assessment, but they generally rely on predefined incisal landmarks or selected tooth pairs. Most previously described digital approaches have preserved this same geometric concept, transferring conventional overjet measurement to three-dimensional dental models and primarily evaluating its accuracy and reproducibility [10,11,12]. 3D Clinical Overjet differs conceptually in that it does not preserve a predefined tooth pair or landmark configuration; instead, it searches across the anterior dental region for the minimum sagittal distance between opposing dental surfaces. Its distinguishing feature therefore lies not in the use of three-dimensional models per se, but in changing the geometric target of the measurement. This surface-based approach may allow for the description of anterior sagittal relationships that vary according to tooth position across the dental arch; however, it also requires dedicated software and a manual search procedure that may increase operator dependency. Accordingly, the present findings should not be interpreted as demonstrating greater accuracy, efficiency, or clinical utility compared with conventional approaches. The objective of the present study was not to demonstrate the superiority of the proposed method over conventional overjet measurement, nor to introduce a new diagnostic parameter. Rather, its purpose was to describe a standardized measurement protocol and document its application in a clinical sample, thereby providing the basis for a subsequent process of methodological validation [17,18,19,20].

Application of the method to the study sample made it possible to document its feasibility and observed behavior under the investigated clinical conditions. The minimum three-dimensional distance could be identified in all subjects at T0 and in 41 of 42 subjects at T1, and its location was not consistently associated with the same pair of incisors. Furthermore, in some patients, its location changed between T0 and T1, suggesting that the measurement obtained using this method may vary in response to dentoalveolar changes induced by orthodontic preparation. This observation does not allow any biological significance to be attributed to the method but demonstrates that the protocol is capable of describing a potentially dynamic anatomical characteristic.

The comparison between 3D Clinical Overjet and conventional radiographic overjet at T0 should likewise be interpreted within the methodological context of the present study for explanatory purposes. The aim was not to demonstrate that the two measurements describe different parameters or to establish which method is more accurate, but rather to determine whether the new procedure exhibited behavior different from that of the conventional measurement. Accordingly, the absence of a significant correlation observed in the present sample should be regarded as a descriptive finding that is useful for generating new research hypotheses but it does not constitute evidence that the two measurements describe different or complementary anatomical information [17,18].

The biological rationale underlying the development of 3D Clinical Overjet is related to the biomechanics of functional mandibular advancement. Functional appliances maintain the mandible in an advanced position, and Class II correction results from variable combinations of skeletal and dentoalveolar adaptations. Moreover, previous evidence suggests that the magnitude of mandibular advancement may influence dentoalveolar effects, particularly mandibular incisor position [13,15]. These observations provide an indirect biomechanical rationale for considering the anterior sagittal dental relationship when planning mandibular advancement. On this basis, the minimum sagittal distance between opposing anterior dental surfaces was hypothesized to represent a potentially relevant anatomical characteristic. However, the present study does not demonstrate that this measurement corresponds to the amount of achievable mandibular advancement or that it has biological or prognostic significance.

From a clinical perspective, the potential interest of 3D Clinical Overjet may lie in situations in which the anterior sagittal relationship is influenced by changes in tooth position and is therefore not fully represented by a measurement restricted to a predefined pair of incisors [21]. This may occur during orthodontic preparation before functional mandibular advancement, when maxillary expansion, incisor alignment, decompensation, or changes in incisor inclination may modify the relative position of the anterior dental surfaces. In the present sample, the tooth determining the minimum sagittal distance varied among patients and could change between T0 and T1, supporting the potential value of a surface-based assessment for describing such geometric changes.

### Limitations

The main limitation of the present study is the lack of methodological validation. Intra-examiner repeatability, inter-examiner reliability, and measurement error were not evaluated; accordingly, no ICC, Bland–Altman analysis, or other formal assessment of measurement agreement was performed [16,17,18,19,20]. Measurements were performed by a single non-blinded examiner, which may have introduced observer-related bias. Moreover, because 3D Clinical Overjet is identified through a manual search for the minimum sagittal distance across the anterior dental surfaces, the procedure may be operator-dependent. Potential sources of systematic measurement error include construction of the occlusal plane, identification of the region of minimum sagittal proximity, positioning of the measurement sections, and selection of the measurement points. The relatively small sample was pragmatically defined, without an a priori sample size calculation, and included consecutive eligible patients. Although consecutive inclusion limited discretionary case selection, the retrospective design and restriction to growing Class II patients selected for functional treatment may have introduced selection bias and may limit generalizability to Class I, Class III, and other dentofacial or occlusal presentations, as well as to different age groups and clinical conditions. In addition, orthodontic preparation between T0 and T1 was not completely uniform: all patients underwent maxillary expansion, whereas additional incisor alignment or decompensation was performed only when clinically indicated. Therefore, the observed T0–T1 changes cannot be attributed to a single standardized procedure and should be interpreted as exploratory. The present study should thus be regarded as a methodological feasibility proposal rather than as evidence supporting a new diagnostic parameter. Future validation studies should assess intra- and inter-examiner reliability, measurement error and agreement using ICC and Bland–Altman analysis, and evaluate the method across Class I, Class II, Class III, and other different malocclusions and anatomical conditions. Finally, practical aspects of implementation, including measurement time and operator-reported technical difficulties, were not systematically recorded and should be evaluated prospectively.

## 5. Conclusions

The present study describes a standardized three-dimensional method, termed 3D Clinical Overjet, for identifying the minimum sagittal distance between opposing anterior dental surfaces without restricting the measurement to a predefined pair of incisors. In this sample of growing patients with Class II malocclusion, the method was feasible and showed that the tooth determining the minimum sagittal distance varied among patients and could change following orthodontic preparation. 3D Clinical Overjet showed no significant association with conventional radiographic overjet at baseline. Overall, these findings demonstrate the feasibility of a surface-based geometric approach for describing the anterior sagittal interarch relationship.

## Figures and Tables

**Table 1:** Demographic, baseline malocclusion, and 3D Clinical Overjet characteristics of the study sample (N = 42).

	Overall
*n*	42
Sex, *n* (%)	
Female	22 (52.4)
Male	20 (47.6)
Age at T0, years	9.11 [8.36, 10.84]
ANB at T0, °	5.85 [5.23, 7.00]
Wits appraisal at T0, mm	3.00 [2.00, 4.00]
Radiographic overjet at T0, mm	5.90 [4.35, 7.65]
First Contact Tooth (T0), *n* (%)	
11	13 (31.0)
12	9 (21.4)
21	15 (35.7)
22	5 (11.9)
3D Clinical Overjet (T0), mm	0.50 [0.30, 1.97]
First Contact Tooth (T1), *n* (%)	
11	13 (31.7)
12	10 (24.4)
21	11 (26.8)
22	7 (17.1)
3D Clinical Overjet (T1), mm	1.70 [0.60, 4.30]
3D Clinical Overjet change (T1−T0), mm	0.25 [−0.12, 1.85]

Note: Continuous variables are presented as median [interquartile range] and categorical variables as *n* (%). Age at T0 ranged from 6.68 to 16.16 years. 3D Clinical Overjet and tooth-distribution data at T1 were available for 41 subjects.

**Table 2:** Distribution of 3D Clinical Overjet and involved maxillary tooth at T0 and T1 (paired samples, N = 41). All data are shown as median (IQR) for continuous variables and as *n* (%) for categorical ones.

	T0	T1	*p*
N	41	41	
Tooth, *n* (%)			0.751
11	12 (29.3)	13 (31.7)	
12	9 (22.0)	10 (24.4)	
21	15 (36.6)	11 (26.8)	
22	5 (12.2)	7 (17.1)	
Clinical OVJ, mm	0.60 [0.10, 2.00]	1.70 [0.60, 4.30]	0.054

Continuous variables are presented as median [IQR] and categorical variables as *n* (%). 3D Clinical Overjet values at T0 and T1 were compared using the Wilcoxon signed-rank test for paired samples. Changes in the distribution of the maxillary tooth involved in the minimum sagittal distance were assessed using a marginal homogeneity test for paired categorical data.

## Data Availability

Data will be available on reasonable request from corresponding author.
